# Who benefits from active music engagement during cancer treatment? Associations of sociodemographic characteristics and risk factors with moderators of intervention effects

**DOI:** 10.3389/fpsyg.2025.1550051

**Published:** 2025-07-29

**Authors:** Steven J. Holochwost, Elizabeth Harman, Kristin Stegenga, Seethal A. Jacob, Sheri L. Robb

**Affiliations:** ^1^Department of Psychology, Lehman College, City University of New York, New York, NY, United States; ^2^School of Nursing, Indiana University, Indianapolis, IN, United States; ^3^Children's Mercy Hospital, Kansas City, MO, United States; ^4^School of Medicine and Riley Children's Health, Indiana University, Indianapolis, IN, United States

**Keywords:** music therapy, active music engagement, pediatric cancer, traumatic stress, sociodemographic risk

## Abstract

Young children and their parents experience frequent and repeated exposure to potentially traumatic events during treatment for cancer. Active Music Engagement (AME) is a dyadic music therapy intervention that has been found to mitigate traumatic stress symptoms among parents who screened high for traumatic stress symptoms (TSS) and reported higher levels of child distress with cancer-related hospitalizations/treatment (child distress). The current study examined sociodemographic characteristics and risk factors that were associated with higher levels of parent TSS and child distress as a means to identify families that may benefit most from AME in the future. Data were collected from the parents (or guardians) of *N* =136 young children (*M*_age_ = 4.88 years, *SD* = 1.56 years, 44.1% female) who were undergoing treatment for cancer. Parents (or guardians) completed measures that captured child and respondent demographics as well as levels of parent TSS and child distress. A series of multivariable linear regression models revealed that poorer child health was associated with significantly higher levels of child distress and parent TSS, while more frequent participation in religious or spiritual practice were associated with lower levels of TSS. Higher levels of cumulative risk were associated with higher levels of child distress and parent TSS, but different, specific risk factors were more strongly associated with each of these outcomes: income-to-needs ratios below the federal poverty level (FPL) were associated with higher child distress, whereas single partner status and parental unemployment were associated with higher levels of parent TSS.

## Introduction

During treatment for cancer, young children and their parents face frequent and repeated exposure to potentially traumatic events, which are defined as events that pose a threat to one's life and/or that evoke fear, horror, and helplessness (De Young et al., 2021; Graf et al., 2013; Kazak and Baxt, 2007; Pai and Kazak, 2006; Price et al., 2016). Due to their developmental stage, young children have an elevated risk for experiencing cancer as traumatic and their reliance on their parents to regulate the emotions raised by these experiences places added physical and emotional strain on their parent (Bates et al., 2022; De Young et al., 2021; Harper et al., 2013; Kazak and Baxt, 2007; Norberg and Boman, 2013). Young child and parent distress is interrelated and prevalent among families with children undergoing cancer treatment, with 40% to 83% of parents reporting traumatic stress symptoms (TSS) within the first month of their child's diagnosis (Bakula et al., 2020; Price et al., 2016; Rodriguez et al., 2011; Woolf et al., 2016). For some parents TSS decreases over time, but for others symptoms persist and are severe (18–33% at 6 months; 7–27% at >10 months post diagnosis) (Bemis et al., 2015; Bruce, 2006; Pai et al., 2007; Price et al., 2016; Woolf et al., 2016). Lowering parent distress and TSS during treatment is associated with lower child distress, improved health-related quality of life, and better psychosocial adjustment after treatment ends (Landolt et al., 2012; Nakajima-Yamaguchi et al., 2016; Pierce et al., 2017). Yet, few empirically validated interventions are available for parents and fewer still address the interrelated nature of child/parent distress (Bradt et al., 2021; Facchini and Ruini, 2021; Koyu and Törüner, 2023; Robb and Hanson-Abromeit, 2014; Rodríguez-Rodríguez et al., 2022; Tang et al., 2020).

Interactive or active music making has been used to address a wide range of developmental and health outcomes in young children across a variety of community and healthcare contexts. Although young children can benefit from receptive experiences like music listening (Nguyen et al., 2010), most music intervention studies have focused on active music making experiences (Facchini and Ruini, 2021; Rodríguez-Rodríguez et al., 2022; González-Martín-Moreno et al., 2021). The hands-on, creative, and improvisatory qualities of making music can provide a developmentally appropriate and immersive experience that supports self-expression, social connectedness, and emotion regulation (O'Callaghan et al., 2011; Harman and Shoemark, 2024; Silberstein et al., 2024; Uggla et al., 2016, 2018). In the last 10 years, there has been an increased number of studies examining the use of interactive music experiences to address the interrelated needs of the child with cancer, their parents (or caregiver), and their larger family system (Boyde et al., 2024; Giordano et al., 2021; Harman and Shoemark, 2024; Heiderscheit, 2022; Robb et al., 2023a; Silberstein et al., 2024; Uggla et al., 2019).

Active Music Engagement (AME) is a theoretically grounded dyadic intervention that uses music-based play to lower the interrelated distress experienced by young children and parents during cancer treatment (see Robb et al., 2023a). Prior studies found that AME not only improved child engagement and coping-related behaviors (Robb et al., 2008; Robb, 2000; Robb et al., 2017), but also mitigated TSS among parents who screened high for traumatic stress and reported higher levels of child distress with cancer-related hospitalizations/treatment (which we refer to as “child distress” in the remainder of the paper) (Robb et al., 2023b). Growing availability of music therapy services in US pediatric hospitals supports implementation of AME as an evidence-based intervention for parents at risk for sustained traumatic stress (Biard et al., 2025; Knott et al., 2020). However, understanding which parents are most likely to derive the greatest benefit from AME will be central to developing successful implementation strategies. Given that child distress and TSS moderated the effects of AME in previous trials (Robb et al., 2023b), identifying parents who may report higher levels of distress for their child and TSS for themselves would be an important first step in this effort.

Ecological systems theory (Bronfenbrenner, 1977; Bronfenbrenner and Morris, 2006) suggests that experiences of distress and TSS do not occur in isolation. Rather, the environments that families inhabit, and specifically the extent to which those environments feature sociodemographic risk factors, will influence the incidence and severity of these experiences. Moreover, the concept of cumulative environmental risk suggests that it may not be the presence or absence of a specific risk factor, but rather their accumulation that influences these experiences (Appleyard et al., 2005; Evans et al., 2013), although some risk factors may exhibit stronger associations with distress and TSS than others (Brown et al., 2021). Research on the association of sociodemographic risk factors and TSS in parents of children with cancer is limited, but emerging. Studies in the United States have focused on mothers of children with cancer and found TSS to be associated with partnership status (single vs. partnered), income/socioeconomic status, and education level (Bemis et al., 2015; Greening et al., 2017).

Both ecological systems theory (Bronfenbrenner, 1977; Bronfenbrenner and Morris, 2006) and the integrative model (García Coll et al., 1996) indicate that sociodemographic characteristics, including age, sex, and, in particular, race and ethnicity, can influence families' experiences in the environments they inhabit, and, thereby, their experiences of distress and TSS. Thus, identifying sociodemographic characteristics, as well as sociodemographic risk factors, associated with higher levels of child distress and TSS in parents during cancer treatment would allow us to more readily identify families that may benefit most from AME. This would have implications for participant screening, recruitment, and clinical service delivery. Therefore, in the current study we examined the associations of child distress (with cancer-related hospitalizations/treatment) and parent traumatic stress symptoms with: (1) child and parent (or guardian) sociodemographic characteristics, and (2) sociodemographic risk factors.

## Method

This secondary analysis used data collected during a multisite trial examining mediators and moderators of AME, a music-based intervention designed to lower distress and improve health outcomes in children (ages 3–8 years) with cancer and their parents (R01NR1578). The Institutional Review Board of Indiana University (study identification #1511888386) approved all data collection procedures. For this study, we examined moderators of AME effects for parent TSS identified in the main trial (Robb et al., 2023a), along with sociodemographic information. Moderators included parent scores on a traumatic stress screener (PCL-6; Lang et al., 2012) and parent-reports of child distress with cancer-related hospitalization/treatment (PIES; Phipps et al., 2005). These measures and the sociodemographic information were collected at baseline, following informed consent. To be included in the main trial, the parent had to be (1) the primary caregiver of a child (ages 3–8 years) undergoing active cancer treatment, (2) at least 18 years of age at the time of consent, (3) able to be present for all intervention sessions, and (4) able to understand English. Active cancer treatment was defined as an expected treatment course of at least 3 days to receive moderate to high intensity chemotherapy (inpatient or outpatient).

### Participants

Participants were *N* = 136 children (*M*_age_ = 4.88 years, *SD* = 1.56 years, 44.1% female) who were undergoing treatment for cancer. In all but six cases, the individual completing the study measures (see below) was the child's mother, stepmother, or foster mother (83.1% of measure respondents) or their father, stepfather, or foster father (12.5%). The remaining six respondents were children's guardians (in four cases) and/or grandparents or great-grandparents. On average, parents and guardians completing the study measures were 34.66 years old (*SD* = 7.00 years).

Table 1a displays the distribution of children as a function of their race and ethnicity. As can be seen in the table, although the majority of children were white (67.6%) and not Hispanic or Latino (15.4%), substantial numbers of children were identified as more than one race (17.6%) or Black or African American (10.3%), and 15.4% were identified as Hispanic or Latino. Similarly, while most parents and guardians identified as White (73.9%) and not as Hispanic or Latino (81.3%), 10.4% identified as Black or African American and 7.5% identified as more than one race and 13.4% of parents and guardians identified as Hispanic or Latino (see Table 1b). For purposes of analyses, child and parent race were coded dichotomously as not minoritized (White) or minoritized (not White). Similarly, child and parent ethnicity were coded as not minoritized (not Hispanic or Latino) or minoritized (Hispanic or Latino).

**Table 1a:** Levels of child distress with prior hospitalizations and parent traumatic stress as a function of child sociodemographic characteristics.

**Disaggregated characteristics**					**Aggregated characteristics**				
	**Child distress**		**Parent traumatic stress**			**Child distress**		**Parent traumatic stress**	
**Child age (*****n*** = **136)**	***r***	***p***	***r***	***p***		***r***	***p***	***r***	***p***
	0.01	0.912	−0.12	0.154					
**Child sex (*****n*** = **136)**	***M***	***SE***	***M***	***SE***	**Child sex (*****n*** = **136)**	***M***	***SE***	***M***	***SE***
- Male (*n* = 76, 56%)	22.90	0.78	13.64	0.60					
- Female (*n* = 60, 44%)	21.92	0.95	13.22	0.73					
**Child race (*****n*** = **136)**					**Child race (*****n*** = **136)**				
- AI/AN (*n* = 1, 1%)	30.00	—	15.00	—	Not minoritized (*n* = 92, 68%)	22.51	0.74	13.48	0.55
- Asian (*n* = 3, 2%)	21.67	5.49	9.67	2.19	Minoritized (*n* = 44, 32%)	22.37	1.07	13.41	0.87
- Black/AA (*n* = 14, 10%)	21.81	2.18	14.50	1.87					
- Native Hawaiian/PI (*n* = 0, 0%)	—	—	—	—					
- White (*n* = 92, 68%)	22.51	0.74	13.48	0.55					
- More than one race (*n* = 24, 18%)	22.38	1.36	13.46	1.10					
- Other (*n* =2, 2%)	23.50	5.50	10.00	2.00					
**Child ethnicity (*****n*** = **136)**					**Child ethnicity (*****n*** = **131)**^1^				
- Hispanic or Latino (*n* = 21, 15%)	22.43	1.35	14.71	1.38	Not minoritized (*n* = 110, 84%)	22.27	0.69	13.20	0.49
- Not Hispanic/Latino (*n* = 110, 81%)	22.27	0.69	13.20	0.49	Minoritized (*n* = 21, 16%)	22.43	1.36	14.71	1.38
- Unknown (*n* = 5, 4%)	27.00	2.14	13.80	3.15					
**Child health (*****n*** = **134)**					**Child health (*****n*** = **134)**				
- Very good (*n* = 6, 5%)	22.67	4.75	11.67	1.48	- (Very) good (*n* = 100, 75%)	21.59^*^	0.75	12.77^*^	0.49
- Good (*n* = 94, 70%)	21.52	0.74	12.84	0.52	- (Very) poor (*n* = 34, 25%)	25.03^*^	0.89	15.38^*^	1.05
- Poor (*n* = 28, 21%)	25.18	1.02	15.00	1.19					
- Very poor (*n* = 6, 5%)	24.33	1.73	17.17	2.29					

1 Respondents whose data were missing or who indicated “unknown” were excluded from these analyses.

AI/AN, American Indian/Alaskan Native; AA, African American; PI, Pacific Islander.

* Between-group difference is significant (*p* < 0.05).

**Table 1b:** Levels of distress with prior hospitalizations and post-traumatic stress as a function of parent sociodemographic characteristics.

**Disaggregated characteristics**					**Aggregated characteristics**				
	**Child distress**		**Parent traumatic stress**			**Child distress**		**Parent traumatic stress**	
**Parent age (*****n*** = **134)**^1^	***r***	***p***	***r***	***p***		***r***	***p***	***r***	***p***
	0.10	0.255	−0.20^*^	0.022					
**Parent race (*****n*** = **134)**^1^	***M***	***SE***	***M***	***SE***	**Parent race (*****n*** = **132)**^1, 2^	***M***	***SE***	***M***	***SE***
- AI/AN (*n* = 2, 2%)	32.00	2.00	20.00	5.00	Not minoritized (*n* = 99, 74%)	22.45	0.71	13.49	0.54
- Asian (*n* = 5, 4%)	22.60	3.44	11.80	1.88	Minoritized (*n* = 33, 26%)	22.03	1.26	13.61	1.01
- Black/AA. (*n* = 14, 10%)	21.29	2.00	14.79	1.79					
- Native Hawaiian/PI (*n* = 0, 0%)	—	—	—	—					
- White (*n* = 99, 74%)	22.45	0.71	13.49	0.54					
- More than one race (*n* = 10, 8%)	20.50	2.11	12.30	1.53					
- Other (*n* = 2, 2%)	26.50	2.50	10.00	2.00					
- Missing (*n* = 2, 2%)^2^	—	—	—	—					
**Parent ethnicity (*****n*** = **134)**^1^					**Parent ethnicity (*****n*** = **127)**^1, 3^				
- Hispanic/Latino (*n* = 18, 13%)	22.67	1.56	15.50^†^	1.50	Not minoritized (*n* = 109, 86%)	22.15	0.68	13.17^†^	0.49
- Not Hispanic/Latino (*n* = 109, 81%)	22.15	0.68	13.17^†^	0.49	Minoritized (*n* = 18, 14%)	22.67	1.56	15.50^†^	1.50
- Unknown (*n* = 7, 5%)^3^	—	—	—	—					
**Parent health (*****n*** = **134)**^1^					**Parent health (*****n*** = **134)**^1^				
- Very good (*n* = 62, 46%)	8.98	0.39	21.42	0.78					
- Good (*n* = 68, 51%)	10.01	0.34	23.50	0.94					
- Poor (*n* = 4, 3%)	9.50	2.96	19.25	2.75					
- Very poor (*n* = 0)	—	—	—	—					
**Parent religious preference (*****n*** = **133)**^1^					**Parent religious preference (*****n*** = **133)**^1^				
- Buddhist (*n* = 1, 1%)	12.00	—	8.00	—	- Preference (*n* = 103, 77%)	21.88	0.69	13.00^†^	0.51
- Christian, not catholic (*n* = 53, 40%)	22.40	0.94	13.28	0.73	- No preference (*n* = 30, 23%)^4^	23.87	1.26	15.17^†^	1.09
- Christian, Catholic (*n* = 42, 32%)	21.02	1.11	12.45	0.70					
- Islam (*n* = 1, 1%)	24.00	—	9.00	—					
- Jewish (*n* = 0, 0%)	—	—	—	—					
- No Preference (*n* = 29, 22%)	23.90	1.30	15.00	1.12					
- Other (*n* = 7, 5%)	24.43	2.27	16.43	2.89					
**Parent participation in religious or spiritual practice (*****n*** = **134)**^1^	***M***	***SE***	***M***	***SE***	**Parent participation in religious or Spiritual practice (*****n*** = **132)**^1^	***M***	***SE***	***M***	***SE***
- Inactive (*n* = 27, 20%)	22.19	1.25	22.19	1.25	- Inactive/Infrequent (*n* = 68, 52%)^5^	22.21	0.82	14.04^†^	0.65
- Infrequent (*n* = 31, 23%)	21.39	1.20	21.39	1.20	- Occasional/Regular (*n* = 64, 48%)	22.65	0.91	12.22^†^	0.58
- Occasional (*n* = 20, 15%)	23.50	1.71	23.50	1.71					
- Regular participant (*n* = 42, 31%)	22.07	1.09	22.07	1.09					
- Not applicable (*n* = 14, 10%)	24.57	2.13	24.57	2.13					
**Household size (*****n*** = **134)**^1^	***r***	***p***	***r***	***p***	**Household size (*****n*** = **134)**^1^	***r***	***p***	***r***	***p***
	−0.03	0.738	−0.08	0.356					
**Household structure (*****n*** = **136)**	***M***	***SE***	***M***	***SE***	**Household structure (*****n*** = **136)**	***M***	***SE***	***M***	***SE***
- Two parents (*n* = 93, 68%)	22.72	0.73	13.38	22.72	- Two parents (*n* = 101, 74%)^6^	23.03	0.73	13.64	0.56
- Father (*n* = 4, 3%)	21.00	4.26	11.75	21.00	- Single parent (*n* = 35, 26%)^7^	20.83	1.03	12.91	0.80
- Mother (*n* = 23, 17%)	20.39	1.19	13.65	20.39					
- Other (*n* = 16, 12%)	24.31	2.11	14.06	24.31					

^1^Respondents included all individuals who were the child's parent [mothers (all types), fathers (all types)] or guardian, regardless of their relationship to the child.

^2^Respondents whose data were missing or who indicated “unknown” were excluded from these analyses.

^3^Respondents whose data were missing or who indicated “unknown” were excluded from these analyses.

^4^No Preference includes one individual who indicated “Other” and then “Secular.”

^5^Includes individuals responding “Not Applicable” if their response to religious affiliation was “No Preference” or “Other, Secular.”

^6^Two parents included any living arrangement in which two adults were present (e.g., mother and step-father, aunt and uncle).

^7^Single parent included any living arrangement in which the child spent their time with one adult at a time (e.g., split custody).

AI/AN, American Indian/Alaskan Native; AA, African American; PI, Pacific Islander.

† Between-group difference approaches significance (*p* < 0.10).

* Bivariate correlation is significant (*p* < 0.05).

### Measures

As noted above, children's parents (or guardians) completed a series of measures at baseline, including a family information form that captured child and respondent demographics, as well as child and respondent health, religion and religiosity, and household size and structure, and the PIES (Phipps et al., 2005) and PCL-6 (Lang et al., 2012). Each item for the PIES was answered on a five-point scale, with higher scores indicating higher levels of distress. Following the scoring guidelines for the PIES, child and parent distress scores were calculated as the sum of items 1 to 8 and 9 to 11, respectively. Each item for the PCL-6 was also answered on a five-point scale (Not at all = 1 to Extremely = 5) and an overall severity score was calculated as the sum of item responses (see Robb et al., 2023b; Supplementary Table 2 for the psychometric properties of this measure). An overall severity score of 14 or more indicates a high level of symptoms, and in this trial such a score triggered a referral to a social worker for additional support.

Four items from the family information form assessed factors that have commonly been used as indicators of sociodemographic risk in studies of pediatric cancer (e.g., Bemis et al., 2015) and in the developmental science literature more broadly (e.g., Burchinal et al., 2008): parental education, parental partner status, parental employment, and household income. The distribution of parents' and guardians' responses for each of these items is displayed in Table 2. Responses for each item were dichotomously recoded as indicative of sociodemographic risk or not based on thresholds used in prior research (Gassman-Pines and Yoshikawa, 2006; Holochwost et al., 2016). Thus, parental or guardian education of high school or less was coded as indicative of risk, as was single partner status and unemployment.

**Table 2:** Levels of distress with prior hospitalizations and post-traumatic stress as a function of sociodemographic risk.

**Disaggregated risk factors**					**Dichotomized risk scores**				
	**Child distress**		**Parent traumatic stress**			**Child distress**		**Parent traumatic stress**	
**Parental education (*****n*** = **134)**^1^	***M***	***SE***	***M***	***SE***	**Parental education (*****n*** = **134)**^1^	***M***	***SE***	***M***	***SE***
6th grade or less (*n* = 2, 2%)	30.00	1.00	16.00	30.00	- No risk (*n* = 128, 96%)	22.28	0.62	13.50	0.48
- 7th−9th grade (*n* = 0, 0%)	—	—	—	—	- Risk (*n =* 6, 4%)	25.17	3.10	13.83	1.91
- 9th−12th grade (*n* = 4, 3%)	22.75	4.25	12.75	22.75					
- High school (*n* = 28, 21%)	22.14	1.26	14.79	22.14					
- Some college (*n* = 36, 27%)	23.39	1.16	14.31	23.39					
- College (*n* = 45, 34%)	19.98	0.87	12.62	19.98					
- Gradudate/Professional (*n* = 19, 14%)	25.84	1.95	12.16	25.84					
**Parent marriage (*****n*** = **134)**^1^					**Parent partner status (*****n*** = **134)**^1^				
- Single (*n* = 28, 21%)	22.32	1.50	15.61	1.10	- No Risk (*n* = 106, 79%)	22.43	0.66	12.96^*^	0.50
- Married (*n* = 86, 64%)	22.92	0.76	13.58	0.57	- Risk (*n =* 28, 21%)	22.32	1.50	15.61^*^	1.10
- Life partners (*n* = 3, 2%)	21.67	3.93	9.67	1.76					
- Divorced/Separated (*n* = 17, 13%)	20.12	1.18	10.41	0.93					
- Widowed (*n* = 0, 0%)	—	—	—	—					
**Parent employed (*****n*** = **132)**^1^					**Parent employment (*****n*** = **132)**^1^				
- Employed full-time (*n* = 46, 35%)	22.61	1.18	12.50	0.72	- No Risk (*n* = 106, 80%)	22.30	0.88	12.45^*^	0.54
- Employed part-time (*n* = 21, 16%)	21.62	1.10	12.33	0.74	- Risk (*n =* 28, 20%)	22.48	0.84	14.46^*^	0.75
- Retired (*n* = 0, 0%)	—	—	—	—					
- Not employed (*n* = 65, 49%)	22.48	0.84	14.46	0.75					
**Household income (*****n*** = **125)**^2^					**Income to needs ratio (*****n** =* **124)**				
- Less than $5,000 (*n* = 8, 6%)	23.75	2.02	16.13	2.30	- No risk (*n* = 89, 72%)	21.97	0.77	13.13	0.56
- $5,000 to $9,999 (*n* = 13, 10%)	22.15	2.02	14.23	1.41	- Risk (*n =* 35, 28%)	24.11	1.15	14.03	0.85
- $10,000 to $14,999 (*n* = 4, 3%)	23.00	3.49	14.50	1.85					
- $15,000 to $19,999 (*n* = 2, 2%)	30.50	12.50	14.50	6.50					
- $20,000 to $24,999 (*n* = 5, 4%)	22.00	3.73	11.20	1.53					
- $25,000 to $34,999 (*n* = 11, 9%)	23.91	1.98	13.09	1.88					
- $35,000 to $49,999 (*n* =10, 8%)	20.30	1.50	14.00	1.61					
- $50,000 to $100,000 (*n* = 42, 34%)	22.12	1.09	13.69	0.94					
- More than $100,000 (*n* = 30, 24%)	22.87	1.47	12.13	0.62					

^1^Respondents included all individuals who were the child's parent [mothers (all types), fathers (all types)] or guardian, regardless of their relationship to the child.

^2^Respondents included all individuals who were the head of the child's household [mothers (all types), fathers (all types)], and guardians, regardless of their relationship to the child.

* Between-group difference is significant at *p* < 0.05.

Prior to dichotomization, household income was recoded as income-to-needs ratios (McLoyd, 1998). This was accomplished by taking the mid-point of the range respondents used to indicate household income (e.g., $7,500 for the range “$5,000 to $9,999”) or the minimum (“less than $5,000”) or maximum values (“more than $100,000”) when those were endorsed and then dividing that figure by the federal poverty level (FPL) for a household corresponding to the reported size for the year in which each family participated in the study. Thus, a family with an income corresponding precisely to the FPL would have an income-to-needs ratio (INR) of 1.0. An INR < 1.0 is indicative of sociodemographic risk (Gassman-Pines and Yoshikawa, 2006).

The four dichotomously coded risk factors were summed to yield a cumulative sociodemographic risk score for each family that provided data on all four risk factors (Burchinal et al., 2000). The mean level of cumulative risk was 1.03 (*SD* = 1.00). Forty-four families (35.2% for whom risk scores could be calculated) had a risk score of 0; the remaining families had scores of 1 (*n* = 48, 38.4%), 2 (*n* = 19, 15.2%), 3 (*n* = 13, 10.4%), or 4 (*n* = 1, 0.8%).

### Data analyses

In the first, preliminary step of our analyses we examined indices of central tendency and variation in measures of child distress with prior hospitalization and parent traumatic stress scores. Given that both ecological systems theory (Bronfenbrenner and Morris, 2006) and the integrative model (García Coll et al., 1996) suggest that levels of cumulative sociodemographic risk may differ among members of minoritized racial and/or ethnic groups and their non-minoritized counterparts, we examined levels of cumulative risk as a function of minoritized racial and ethnic group identity to determine whether subsequent analyses in which cumulative risk was the focal predictor should include minoritized racial and/or ethnic group identity.

**Figure 1:**
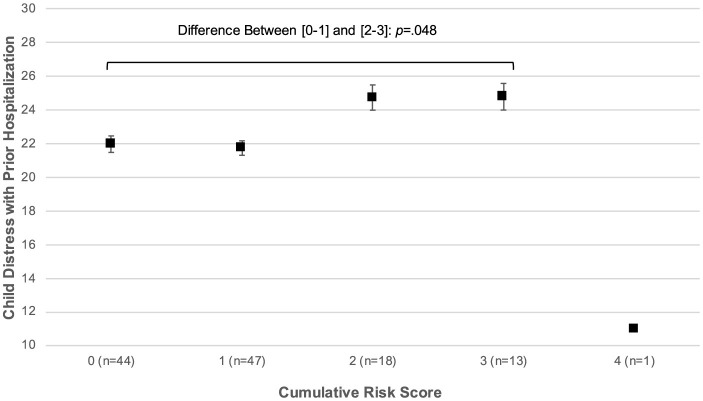
Association between cumulative sociodemographic risk and child distress with prior hospitalizations.

**Figure 2:**
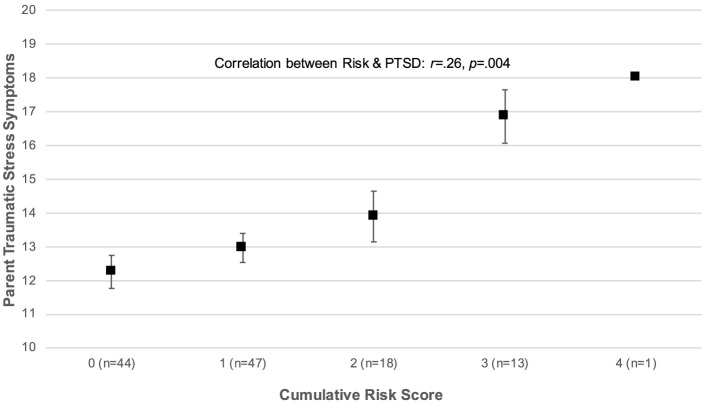
Association between cumulative sociodemographic risk and parent traumatic stress symptoms.

To examine the associations of child distress and parent traumatic stress with sociodemographic characteristics we first examined mean levels of distress as a function of each sociodemographic characteristic that was coded categorically (e.g., child race) and the bivariate correlation between levels of distress and each continuous characteristic (e.g., child age). All sociodemographic characteristics included in our analyses are listed in Table 3. A series of four multivariable linear regression models were then estimated to examine the unique associations of sociodemographic characteristics with levels of child distress (Models 1a and 1b) and parent traumatic stress symptoms (Models 2a and 2b). Each model included as independent variables the sociodemographic characteristics identified via the preliminary analyses as being associated with the dependent variable in question at a rate achieving or approaching significance (*p* < 0.10). To reduce the standard errors for each correlation coefficient, the aggregated versions of these characteristics were included rather than the disaggregated versions. The final model in each set included cumulative risk as a covariate in the form suggested by the nature of the association between cumulative risk and child distress or parent traumatic stress (see Figures 1, 2 below).

**Table 3:** Sociodemographic characteristics and risk factors included in analyses.

**Characteristics**	**Risk factors**
**Child characteristics**	
- Age	- Parent education
- Sex	- Parent marital status
- Race	- Parent employment
- Ethnicity	- Household income-to-needs ratio
- Health	
**Parent characteristics**	
- Age	
- Race	
- Ethnicity	
- Health	
- Religion	
- Religiosity	
**Household characteristics**	
- Size	
- Structure	

The associations of child distress and parent traumatic stress with sociodemographic risk were investigated in a similar fashion. We first examined mean levels of distress and traumatic stress as a function of each sociodemographic risk factor listed in Table 3 is both its original and dichotomized forms, and then examined the distribution of these scores as a function of cumulative sociodemographic risk. Six multivariable regression models were then estimated to examine the associations between sociodemographic risk and child distress (Models 3a through 3c), and between risk and parent traumatic stress symptoms (Models 4a through 4c). The first model in each set (Model a) examined the association between cumulative sociodemographic risk scores and the dependent variable in question; the next model in each set (Model b) examined the association of cumulative risk and the dependent variable while controlling for minoritized race, given that our preliminary analyses revealed a significant association between minoritized race and cumulative risk scores. The final model in each set (Model c) replaced the cumulative risk score with the dichotomized versions of the four risk factors that comprised the score (per Bemis et al., 2015), given that each risk factor may not exhibit an association of equal strength with distress or traumatic stress (Brown et al., 2021).

All analyses were conducted using SPSS (v. 28). Given the very low prevalence of missing data, the essentially descriptive nature of the analyses, and the fact that all other analyses were multivariate regressions (for which listwise deletion is robust to violations of the assumption that data are missing at random; Allison, 2002), missing data were accommodated using listwise deletion.

## Results

### Preliminary results

Parents and guardians (*n* = 134) reported a mean child distress score of 22.49 (*SD* = 6.99) out of a maximum possible score of 45. The average level of TSS was 13.37 (*SD* = 5.40) out of a maximum possible score of 30. Levels of cumulative risk were significantly higher among families in which children were identified as members of minoritized racial groups [*t*_(121)_= 2.78, *p* = 0.006]. A parallel association was not observed for ethnicity (*p* = 0.613).

### Associations of child distress and parent traumatic stress with sociodemographic characteristics

The distributions of child distress scores and TSS by **child** sociodemographic characteristics are displayed in Table 1a. As can be seen in the table, there was no association between these scores and children's age or aggregated race (not minoritized vs. minoritized). However, children who were rated as being in poor or very poor health had significantly higher child distress scores [*t*_(132)_ = −2.49, *p* = 0.014], and parents or guardians of these children reported significantly higher TSS [*t*_(132)_ = −2.50, *p* = 0.014].

The distributions of child distress scores and TSS as a function of **parent or guardian** sociodemographic characteristics are presented in Table 1b. Parents and guardians who were younger reported significantly higher levels of TSS [*r*_(134)_ = −0.20, *p* = 0.022], and parents and guardians who were Hispanic or Latino also reported higher TSS at a rate approaching significance [*t*_(125)_ = −1.67, *p* = 0.098]. Parents and guardians who reported no religious preference reported higher TSS at a rate approaching significance [*t*_(131)_ = 1.95, *p* = 0.053]. Parallel associations were observed when considering the frequency with which parents' and guardians' engaged in their religious or spiritual practice: those who reported that they were inactive or infrequent participants reported higher levels of TSS [*t*_(128)_ = 1.96, *p* = 0.052) at a rate approaching significance when compared to parents and guardians who were occasional or regular participants.

A series of multivariable linear regression models revealed that poorer child health was associated with significantly higher levels of child distress (see Table 4, Model 1a: *B* = 3.44, *SE* = 1.38, *p* = 0.014) and that this association was observed after including cumulative risk as a covariate (Model 1b: *B* = 3.59, *SE* = 1.44, *p* = 0.014). Child health and the frequency of participation in religious or spiritual practice were the most salient sociodemographic characteristics when considering parents' and guardians' TSS. More frequent participation was associated with lower levels of TSS (Model 2a: *B* = –2.00, *SE* = 0.86, *p* = 0.022), and this association held after controlling for levels of cumulative risk (Model 2b: *B* = –2.12, *SE* = 0.89, *p* = 0.019). Poorer child health was also associated with higher levels of TSS before (Model 2a: *B* = 2.30, *SE* = 1.00, *p* = 0.023) and after controlling for risk scores (Model 2b: *B* = 2.70, *SE* = 1.01, *p* = 0.009).

**Table 4:** Regressing levels of child distress with prior hospitalizations and parent traumatic stress symptoms on sociodemographic characteristics.

**Model**	**Independent variables**	**Regression coefficients**		**Model results**		
**Model 1: Child distress with prior hospitalizations**		***B*** **(*****SE)***	***p***	*R* ^2^	***F***	***p***
1a	Child health (1 = poor/very poor)	3.44 (1.38)	0.014	0.045	6.18	0.014
1b	Child health (1 = poor/very poor)	3.91 (1.42)	0.007	0.096	6.33	0.002
	Cumulative risk (1 = 2 or 3 risk factors)	3.59 (1.44)	0.014			
**Model 2: Parent traumatic stress symptoms**		***B*** **(*****SE)***	***p***	*R* ^2^	***F***	***p***
2a	Parent age	−0.10 (0.06)	0.096	0.113	3.84	0.006
	Parent religiosity (1 = occasional/regular)^a^	−2.00 (0.86)	0.022			
	Child ethnicity (1 = minoritized)^b^	1.31 (1.17)	0.266			
	Child health (1 = poor/very poor)	2.30 (1.00)	0.023			
2b	Parent age	−0.09 (0.06)	0.143	0.120	4.17	0.002
	Parent religiosity (1 = occasional/regular)	−2.12 (0.89)	0.019			
	Child ethnicity (1 = minoritized)	1.45 (1.19)	0.224			
	Child health (1= poor/very poor)	2.70 (1.01)	0.009			
	Cumulative risk (continuous)	0.69 (0.48)	0.158			

a When both parent religious preference and parent religiosity were associated with a dependent variable, only the latter was used to avoid collinearity and because the group sizes for religiosity were distributed more even than those for religious preference.

b Child ethnicity was available for a larger number of participants and was therefore used as our measure of ethnicity in all analyses.

### Associations of child distress and parent traumatic stress with sociodemographic risk

Table 2 presents child distress scores and TSS as a function of disaggregated and dichotomized risk factors. Although levels of child distress scores were higher for parents and guardians categorized as being at risk on the basis of their education, partner status, employment, and income-to-need ratios, these differences were not significant. However, TSS were significantly higher among parents and guardians who were categorized as being at risk on the basis of their partner status [*t*_(132)_ = −2.34, *p* = 0.021] or their employment [*t*_(130)_ = −2.19, *p* = 0.030].

Figure 1 displays levels of child distress as a function of cumulative sociodemographic risk. The figure suggests a non-linear association, such that there was a significant difference in child distress scores among families confronting 0 or 1 risk factors, relative to those confronting 2 or 3 factors [*t*_(120)_ = −1.99, *p* = 0.048]. Therefore, subsequent analyses of the association between cumulative risk and child distress used a categorically-recoded risk as the focal predictor (0 = 0 or 1 risk factors; 1 = 2 or 3 risk factors). Figure 1 also revealed the presence of a bivariate outlier in the associations between cumulative risk and child distress as defined by Tukey (1977): the same respondent received a cumulative risk score of 4 but reported relatively low levels of child distress. This individual was excluded from subsequent analyses of the associations between sociodemographic risk and distress, given the potential for bivariate outliers to bias model estimates (Zimmerman, 1994). The distribution of parent and guardian TSS scores as a function of cumulative risk is displayed in Figure 2, and this distribution clearly depicts a positive, linear association between risk and TSS with no evidence of bivariate outliers [*r*_(123)_ = 0.26, *p* = 0.004].

The results of the multivariable linear regression models indicated that although higher levels of categorically-coded cumulative risk scores were associated with significantly higher levels of child distress (see Table 5, Model 3a: *B* = 2.91, *SE* = 1.46, *p* = 0.048), adding minoritized child race as a covariate yielded overall model results that were not statistically significant [Model 3b: *R*^2^ = 0.035, *F*_(2, 119)_ = 2.14, *p* = 0.122]. Replacing cumulative risk scores with the set of four risk factors to predict child distress resulted in a model that accounted for ~10% of the variance in child distress scores [Model 3c: *R*^2^ = 0.099, *F*_(4, 99)_ = 2.72, *p* = 0.034], and families whose income-to-needs ratios were categorized as indicative of risk reported significantly higher levels of child distress after controlling for the categorization of the other three risk factors (*B* = 3.50, *SE* = 1.49, *p* = 0.021).

**Table 5:** Regressing levels of distress with prior hospitalizations and parent traumatic stress symptoms on sociodemographic risk.

**Model**	**Independent variables**	**Regression coefficients**		**Model results**		
**Model 3: Child distress with prior hospitalizations**		***B*** **(*****SE)***	***p***	*R* ^2^	***F***	***p***
3a	Cumulative risk (1 = 2 or 3 risk factors)^a^	2.91 (1.46)	0.048	0.032	3.98	0.048
3b	Cumulative risk (1 = 2 or 3 risk factors)	3.06 (1.49)	0.042	0.035	2.14	0.122
	Child race (minoritized = 1)^b^	−0.81 (1.40)	0.565			
3c	Parental education (risk = 1)	7.60 (4.61)	0.103	0.099	2.72	0.034
	Parental partner status (risk = 1)	−0.12 (1.61)	0.942			
	Parental employment (risk = 1)	0.84 (1.30)	0.520			
	Household income-to-needs ratio (risk = 1)	3.50 (1.49)	0.021			
**Model 4: Parent traumatic stress symptoms**		***B*** **(*****SE)***	***p***	*R* ^2^	***F***	***p***
4a	Cumulative risk (1 = 2 or 3 risk factors)^a^	1.37 (0.45)	0.004	0.060	7.81	0.006
4b	Cumulative risk (1 = 2 or 3 risk factors)	1.37 (0.45)	0.004	0.068	4.37	0.015
	Child race (minoritized = 1)	−0.36 (1.00)	0.719			
4c	Parental education (risk = 1)	1.87 (3.58)	0.603	0.141	4.09	0.004
	Parental partner status (risk = 1)	3.77 (1.24)	0.003			
	Parental employment (risk = 1)	2.73 (1.00)	0.008			
	Household income-to-needs ratio (risk = 1)	−1.06 (1.16)	0.362			

a Cumulative risk was divided into two groups about the median: [0–1] and [2–3].

b Cumulative risk varied significantly as a function minoritized racial status and is therefore included in the model as a covariate.

Higher levels of cumulative risk were also associated with higher levels of TSS (Model 4a: *B* = 1.37, *SE* = 0.45, *p* = 0.004), and this association held after controlling for whether children were members of a minoritized racial group (Model 4b: *B* = 0.44, *SE* = 0.20, *p* = 0.025). Replacing cumulative risk scores with the dichotomized versions of each risk factor resulted in a model that accounted for substantially more variance in TSS scores [Model 4c: *R*^2^ = 0.141, *F*_(4, 100)_ = 4.09, *p* = 0.004]. At risk categorizations of partner status (Model 4c: *B* = −3.77, *SE* = 1.24, *p* = 0.003) and employment (Model 4c: *B* = 2.73, *SE* = 1.00, *p* = 0.008) were each associated with significantly higher TSS scores.

## Discussion

In our primary study, parents who derived the most benefit from an AME intervention reported high distress for their child and elevated traumatic stress symptoms for themselves (Robb et al., 2023b). The purpose of this secondary analysis was to better understand these parents by identifying sociodemographic characteristics and risk factors that were associated with higher levels of child distress and parent TSS. These findings provide additional information to guide subsequent research and the work of pediatric music therapists.

Our examination of **sociodemographic characteristics** showed that parents who rated their child's health as poor reported significantly higher distress in their child and higher TSS for themselves. In addition, these associations were robust to controls for cumulative risk and other sociodemographic characteristics that our preliminary analyses suggested may be related to TSS (i.e., parent age and minoritized child ethnicity). The finding that ***poor child health*** had a significant association with child distress is not surprising, but it is unclear how parents interpreted this question. Parent perceptions of their child's health during cancer treatment is influenced by numerous factors including their prognosis, treatment intensity, the presence or absence of side effects, social support, and cancer-related distress exhibited by their child (Patterson et al., 2004; Price et al., 2016; Schwartz-Attias et al., 2024). The PIES measures a child's distress with cancer and treatment-related experiences such as medical procedures, chemotherapy, hospitalization, and staff relationships (Phipps et al., 2005). As such, we would expect that parents who observe that their child is demonstrating more difficulties with their cancer treatment might also rate their child as having poor health. Importantly, poor child health also had a significant association with parent TSS. These findings are consistent with studies that have established parent perceptions about their child's health and wellbeing as a strong predictor of parent TSS during cancer treatment (Brosbe et al., 2011; Feng et al., 2024; Kahana et al., 2006; Norberg et al., 2012; Price et al., 2016) and offers an additional marker for identifying parents who may benefit from the AME intervention.

We also found that the frequency of parents' ***religious or spiritual practice*** had a significant association with TSS, such that more regular religious or spiritual practice was associated with lower levels of TSS. Researchers have explored the relationship of faith, religiosity, and spirituality to psychological distress. However, religiosity and spirituality are not the same, and neither of these complex, multi-dimensional constructs can be represented by religious practice alone (Villani et al., 2019). That said, participating in religious activities can provide a sense of community, bonding, and support for individuals and families (Dunbar, 2021), as religious communities often provide resources such as food, emotional support, material help, and social networking (Dolan et al., 2021). As such, the frequency of regular religious or spiritual practice may reflect the degree of social support families receive, and social support has been identified as an important factor that contributes to reduced psychological distress in parents during cancer treatment (Feng et al., 2024; Melguizo-Garín et al., 2023, 2022; Sloper, 2000; Schwartz-Attias et al., 2024; Tremolada et al., 2013).

Our analyses of the associations between **sociodemographic risk factors** (education, partner status, employment, and household income-to-needs ratios) and child distress indicated that families who experienced higher levels of **cumulative risk** (a score of 2 our greater) reported more distress in their child, relative to families who experienced lower levels of risk (a score of 1 or lower), and that this association was robust to controls for minoritized race. These findings are consistent with those reported by Bemis et al. (2015), who found that greater sociodemographic disadvantage predicted higher levels of distress for mothers of children with cancer, but that race was not associated with child or parent distress. Our analyses also indicated that including each sociodemographic factor together in one regression model accounted for a larger amount of the variance in child distress scores (~10%) than the cumulative risk score (~4%). In the former model, only **income to needs ratio** was associated with child distress, such that families with household incomes below the federal poverty level (i.e., families in poverty) reported significantly higher child distress. This finding is broadly consistent with the family stress model, which asserts that the stress associated with living in poverty may undermine parents' capacity to provide optimal emotional support for their children (Conger et al., 1994), and the stress of poverty is undoubtedly exacerbated by the economic and emotional burden of having a child with cancer (Bona et al., 2014; Eche-Ugwu et al., 2024).

We also observed a significant association between higher **cumulative risk scores** and higher TSS in parents. As with child distress, this association was robust to controls for minoritized race, and including each factor together in one regression model accounted for a larger portion of the variance in traumatic stress symptoms (~14%) than the cumulative risk score alone (~7%). In the model including each factor simultaneously, **single partner status** and **parental unemployment** were both significantly associated with higher levels of TSS. These findings are also consistent with those reported by Bemis et al. (2015), who found a significant association between single parenthood with greater TSS in parents of children with cancer. Like the frequency of participation in religious or spiritual practice, marital status and employment may signal the importance of social support for parents of children with cancer. Numerous studies (e.g., Klassen et al., 2012; Lundgren et al., 2023), have reported the isolating potential of childhood cancer treatment for parents. This is especially true for young children where one parent may be dedicated to the daily care of their hospitalized child, which may isolate them from family and their social networks, including coworkers. Being an unpartnered parent may compound this sense of isolation and stress (Granek et al., 2014).

This finding underscores the importance of recent studies examining the important role of music as social support for families of young children with cancer (Boyde et al., 2024; Giordano et al., 2021; Harman and Shoemark, 2024; Heiderscheit, 2022; Silberstein et al., 2024; Uggla et al., 2019). Musical engagement in both clinical and community settings has been found to increase social connectedness, decrease social isolation, and support relationships (Bourdaghs and Silverman, 2020; Loi et al., 2022; Murphy and McFerran, 2017). Although we could not identify any pediatric cancer studies that have explored sociodemographic factors associated with benefits from music therapy, a study of adult patients found that terminally ill, non-partnered cancer patients and patients without children reported greater improvements to their mood and feeling understood following a course of music therapy (Kordovan et al., 2016).

This study is not without **limitations**. By their nature, cumulative risk models endorse the concept of equifinality (Evans et al., 2013; Evans, 2003) whereby pathways may lead to the same (or at similar) outcomes (Cicchetti and Rogosch, 1996). However, it is possible that there may be specificity in the associations between risk factors and different outcomes, such that the association between a given risk factor and a given outcome is different in its strength or nature than the association between that risk factor and another outcome. Our results suggest that this is the case for child distress and parent TSS: while levels of cumulative sociodemographic risk predict child distress and TSS, there is also specificity in the associations between sociodemographic risk factors and each of these outcomes.

Moreover, data on each of the sociodemographic characteristics and risk factors included in our analyses were based on parents' or guardians' responses to a single item. While this may be appropriate for certain constructs (e.g., partner status), a single item cannot adequately assess complex, multidimensional constructs such as religious and spiritual practice (Villani et al., 2019). Finally, this analysis used cross sectional data collected during a single time point and does not investigate how these correlations may change over time. We know that distress patterns will change over the cancer treatment trajectory (Galtieri et al., 2024; Price et al., 2016), and as such, repeated and holistic assessment is best practice (Kazak et al., 2015; Pai et al., 2019).

## Conclusions and recommendations

The impact of poverty and social support on child and parent distress is well established. Findings from the current study serve to expand our understanding of parents who reported lower TSS following AME. Our findings suggest that parent perceptions of their child's health are an important driver for both child distress and parent TSS, and AME may be particularly helpful for these families given our finding that changes in parent perceptions of their child's health and wellbeing is a likely mediator of AME effects (Robb et al., 2023b). We also found that families already experiencing sociodemographic disadvantage may derive more benefit from AME than families who present with less disadvantage, but that specific risk factors may differentiate parents who report higher levels of child distress from those who report higher TSS. These findings suggest the benefit of using these particular risk factors when attempting to identify families that may benefit from AME and the use of additional measures to assess these specific risk factors in greater detail. Finally, frequency of religious practice, unemployment, and being a single parent were associated with higher parent TSS and suggest that AME may bring an important form of social support to children and parents.

This is informative to AME implementation studies and clinical practice, and efforts to provide evidence-based care to families. In addition to considering sociodemographic risk factors, and consistent with the Standards for Psychosocial Care in Pediatric Cancer (Knott et al., 2022; Wiener et al., 2015), we recommend repeated screening of child and parent distress to identify changing needs and facilitate prompt referrals for AME and other supportive care services. We also recommend measuring social support as a potential moderator of AME benefit in future trials and the inclusion of modestly expanded item sets to index multidimensional constructs.

## Data Availability

The data analyzed in this study is subject to the following licenses/restrictions. The de-identified data supporting the conclusions of this article can be obtained from the corresponding author via a written data-sharing agreement. Requests to access these datasets should be directed to shrobb@iu.edu.
